# A single molecule investigation of i-motif stability, folding intermediates, and potential as *in-situ* pH sensor

**DOI:** 10.3389/fmolb.2022.977113

**Published:** 2022-08-22

**Authors:** Golam Mustafa, Prabesh Gyawali, Jacob A. Taylor, Parastoo Maleki, Marlon V. Nunez, Michael C. Guntrum, Sajad Shiekh, Hamza Balci

**Affiliations:** Department of Physics, Kent State University, Kent, OH, United States

**Keywords:** i-motif, FRET, förster resonance energy transfer, single molecule, PH sensor, folding intermediates

## Abstract

We present a collection of single molecule work on the i-motif structure formed by the human telomeric sequence. Even though it was largely ignored in earlier years of its discovery due to its modest stability and requirement for low pH levels (pH < 6.5), the i-motif has been attracting more attention recently as both a physiologically relevant structure and as a potent pH sensor. In this manuscript, we establish single molecule Förster resonance energy transfer (smFRET) as a tool to study the i-motif over a broad pH and ionic conditions. We demonstrate pH and salt dependence of i-motif formation under steady state conditions and illustrate the intermediate states visited during i-motif folding in real time at the single molecule level. We also show the prominence of intermediate folding states and reversible folding/unfolding transitions. We present an example of using the i-motif as an *in-situ* pH sensor and use this sensor to establish the time scale for the pH drop in a commonly used oxygen scavenging system.

## Introduction

**FIGURE 1:**
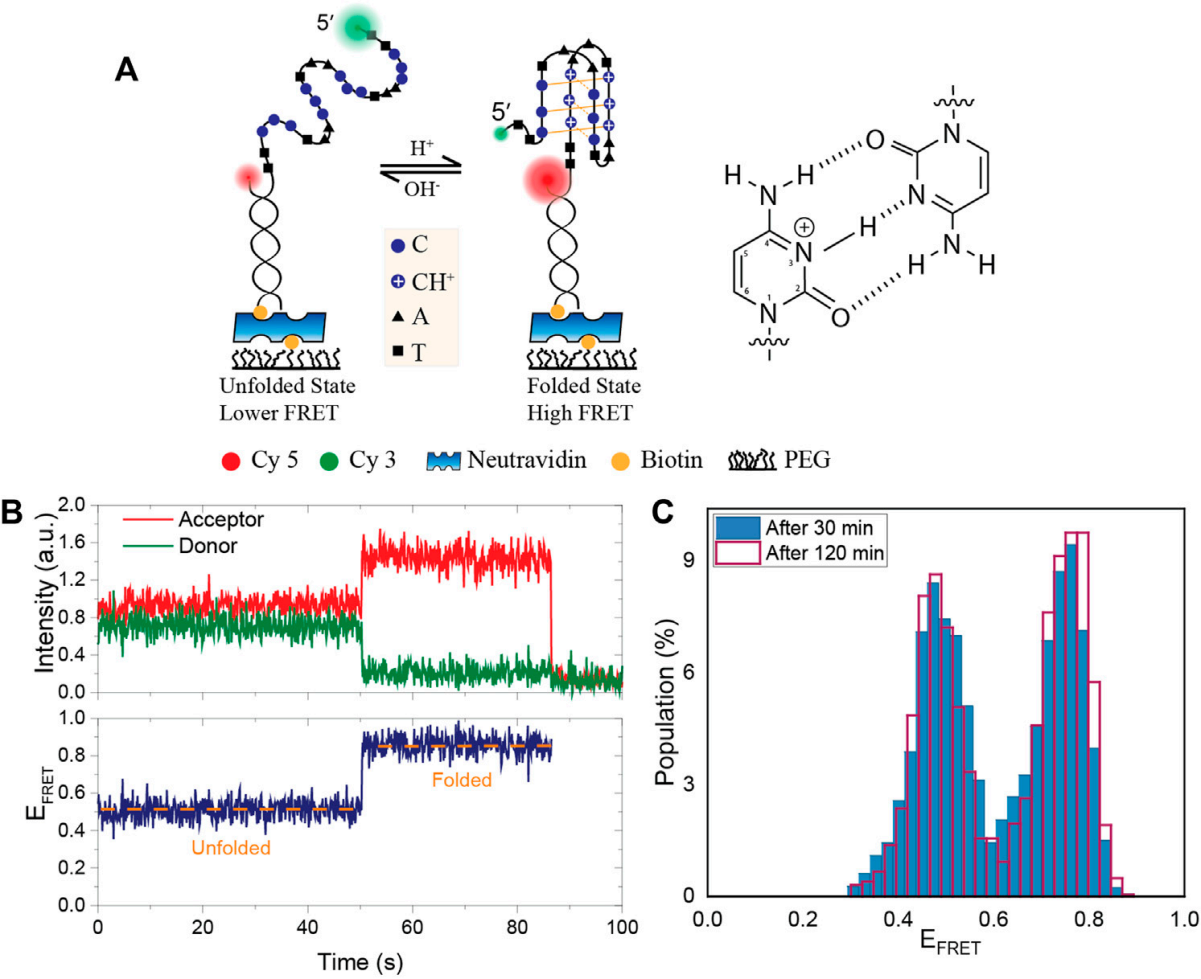
Schematic of the DNA construct, an example smFRET time trace and the stability of pH in PCA/PCD oxygen scavenging system. **(A)** A partial duplex DNA construct were formed by a biotinylated acceptor (Cy5) labeled short strand and a donor (Cy3) labeled long strand that contains an i-motif forming sequence. Folding the DNA into an i-motif brings the donor and acceptor fluorophores closer together, increasing FRET efficiency. Hoogsteen hydrogen bonds between cytosines and protonation of N3 atom are shown in the schematic. **(B)** Representative smFRET trace demonstrating a transition from the unfolded to folded state. **(C)** The FRET distribution at initial pH of 6.3 after 30 min (blue filled bar) and 120 min (red lined bar) incubation of PCA/PCD in the chamber. When PCA/PCD is used as the oxygen scavenging system, the distribution essentially remains unchanged over a 120-min imaging period, implying that the pH remains constant to within ±0.05 of the initial pH.

Nucleic acids can form sequence-dependent secondary structures, such as hairpins, cruciforms, triplex DNA, as well as tetraplex structures, such as the G-quadruplex (GQ) and the intercalated-motif (i-motif). The cytosine rich (C-rich) sequences, that are complementary to GQ forming G-rich sequences, can adopt an i-motif structure which is composed of two parallel duplexes held together by intercalated hemiprotonated cytosine (C:CH^+^) base pairs as well as loop regions composed of bases intervening between the cytosine runs (Gehring et al., 1993; Liu and Balasubramanian, 2003; Liedl and Simmel, 2005; Shu et al., 2005) (Figure 1A). Cytosines, like guanines in the GQ, are base-paired *via* Hoogsteen hydrogen bonds; however, i-motifs form more readily at acidic pH as their formation requires the protonation of cytosines (Choi et al., 2011). Given the abundance of potentially GQ forming sequences in human promoters and telomeres (Chambers et al., 2015), there is an abundance of sites that could form i-motif as well, including promoters of important oncogenes (Manzini et al., 1994).

The formation and stability of the i-motif depends on the environmental conditions such as pH (Jin et al., 2009), cationic effects (Kim et al., 2014; Day et al., 2015; Gao et al., 2016), ionic strength (Mergny et al., 1995; Kim et al., 2014; Nguyen et al., 2017), temperature (Nguyen et al., 2017), and molecular crowding (Cui et al., 2013; Reilly et al., 2015). The stability is also affected by the strand sequence: the melting temperature of an i-motif increases with the number of cytosines in the sequence (McKim et al., 2016; Fleming et al., 2017), and decreases with the length of the loops (Gurung et al., 2015). Epigenetic modification of cytosines within i-motif sequences might also affect stability of i-motif. For example, i-motifs that remained stable at physiological pH levels were reported to be more likely to contain methylated cytosines (Wright et al., 2020).

It has been demonstrated that i-motif structures can be stabilized at physiological pH under molecular crowding conditions (Rajendran et al., 2010; Cui et al., 2013; Li et al., 2016), in the presence of carboxyl-modified single walled carbon nanotubes (Chen et al., 2012), or in the presence of silver(I) cations (Day et al., 2013). Zeraati et al. (Zeraati et al., 2018) found that an antibody fragment called iMab specifically binds to i-motif structures, and that the interaction between iMab and i-motif distinguishes i-motifs from other secondary structures such as GQs. Using this approach, they were able to visualize i-motif structures in telomeres and promoter regions within live human cancer cells, providing significant support for the physiological relevance of these structures. Dzatko et al. used in-cell NMR spectroscopy to evaluate the stabilities of i-motif structures in the complex cellular environment. They demonstrate that i-motifs formed from naturally occurring C-rich sequences in the human genome are stable and persist in human cell nuclei (Dzatko et al., 2018). Tang et al. used immunofluorescence staining with an antibody specific for the endogenous transcription factor BmILF, which binds i-motif structures with high specificity, to visualize i-motif structures in the nuclei and chromosomes of the testis of the invertebrate *Bombyx mori*. They also reported that the number of i-motif structures observed increased as the pH of the genome was changed from basic to acidic (Tang et al., 2020).

Since the formation of C:CH+ base pairs requires protonation of cytosine, the i-motif structure undergoes a sharp transition from folded to random coil (unfolded) conformation at around pH 6.5, below which it remains folded. (Leroy et al., 1993; Ahmed et al., 1994; Liu and Balasubramanian, 2003; Liedl and Simmel, 2005; Shu et al., 2005). The reversible and rapid transformation of the C-rich sequences from unfolded to folded i-motif with pH has potential applications in the development of technologies such as DNA nanomachines (Liu and Balasubramanian, 2003; Liu et al., 2006; Wang et al., 2011), targeted drug delivery systems (Dong et al., 2014), logic operation switches (Miyoshi et al., 2006; Yang et al., 2010; Li et al., 2012), cellular pH sensors (Modi et al., 2009; Surana et al., 2011), and electrochemical sensors for proton detection (Song et al., 2012). It is also possible to tune the pH range that the i-motif can sense and make it a more versatile pH sensor by introducing structural changes within the DNA constructs.

**FIGURE 2:**
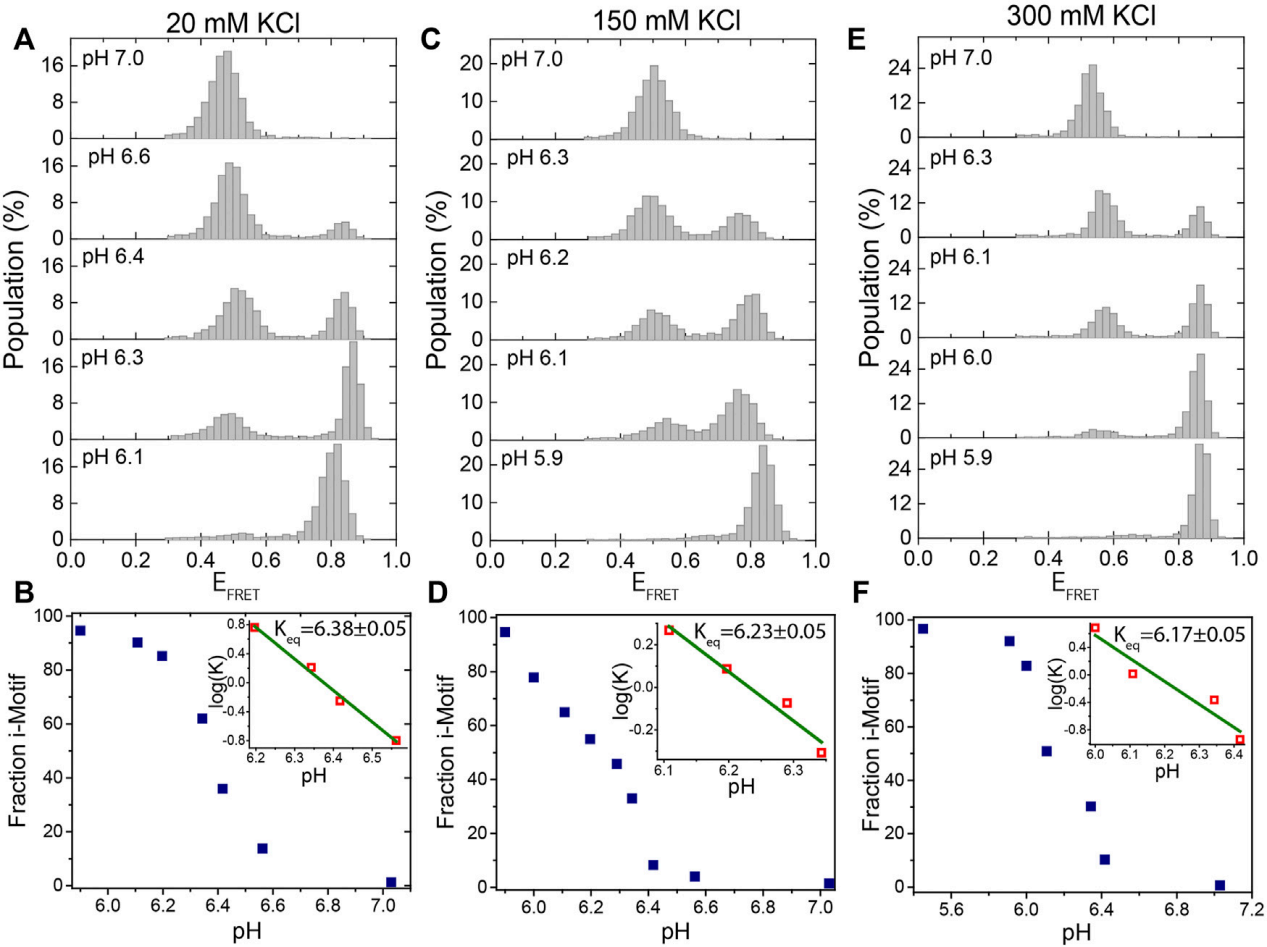
Titration of pH at a fixed KCl concentration. **(A,B)** at 20 mM KCl; **(C,D)** at 150 mM KCl; and **(E)**-**(F)** at 300 mM KCl. The pH was titrated from 7.0 to 5.9 at each of these KCl concentrations. Under all of these ionic conditions, the DNA molecules are exclusively unfolded at pH 7.0 and exclusively folded at pH 5.9. We observe two peaks at intermediate pH levels, around 6.3, which represent the folded or unfolded states. The evolution of the fraction of i-motif molecules as a function of pH is depicted in **(B)**, **(D)**, and **(F)**. The green lines in the inset are linear fits to the most rapidly varying segments of the of the curve of log(K) vs. pH data.

**FIGURE 3:**
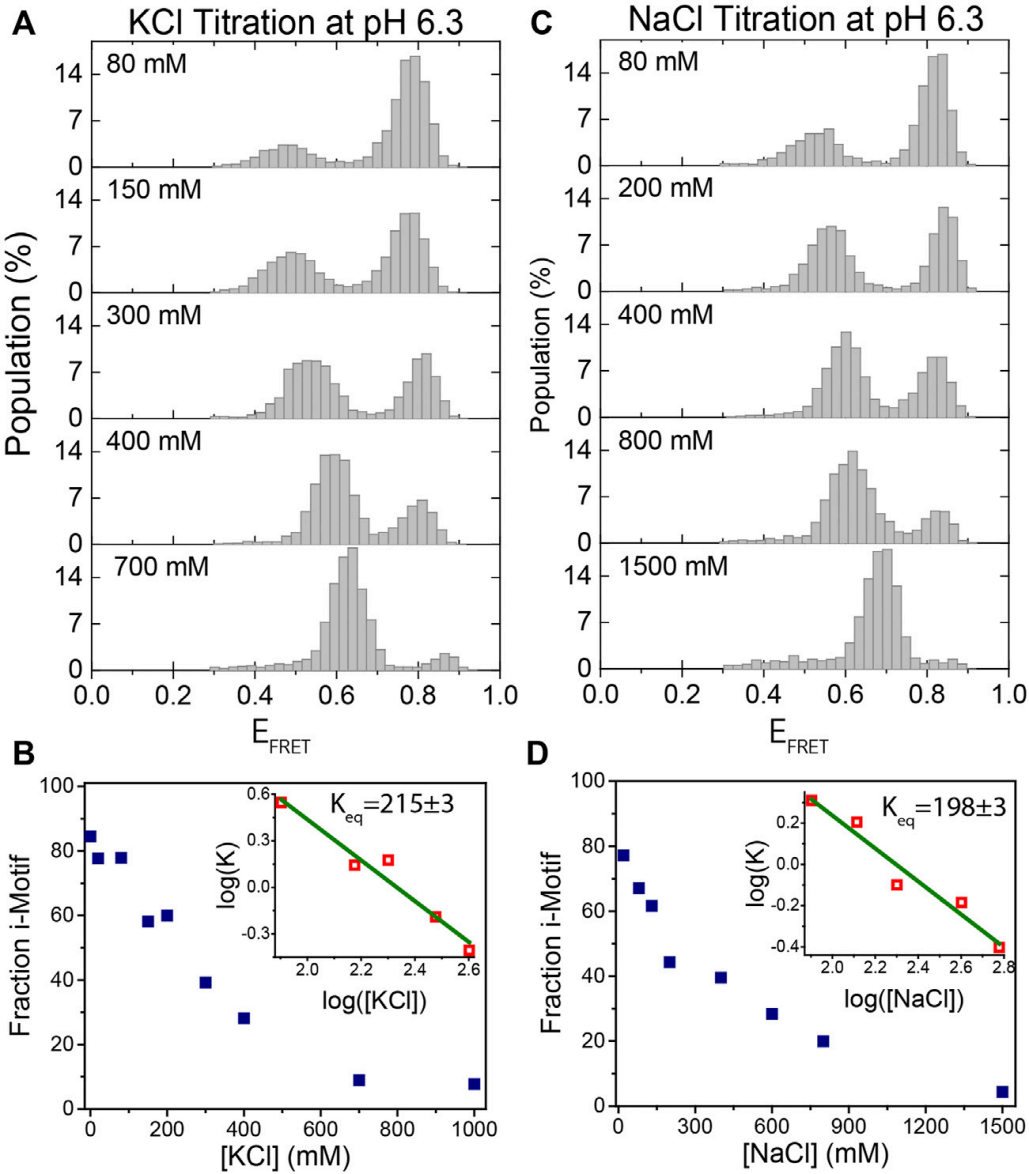
KCl and NaCl titration at pH 6.3. **(A)**–**(B)** KCl titration between 0 and 1,000 mM. **(C)**–**(D)** NaCl titration between 0 and 1,500 mM. The majority of molecules are folded at low salt concentrations and they gradually unfold as the salt concentration increases. The evolution of the fraction of i-motif molecules as a function of (KCl) and (NaCl) are depicted in **(B)** and **(D)** respectively. Green lines in the inset are linear fits to the most rapidly varying segments of the curve of log(K) vs. log(salt concentration) data.

**FIGURE 4:**
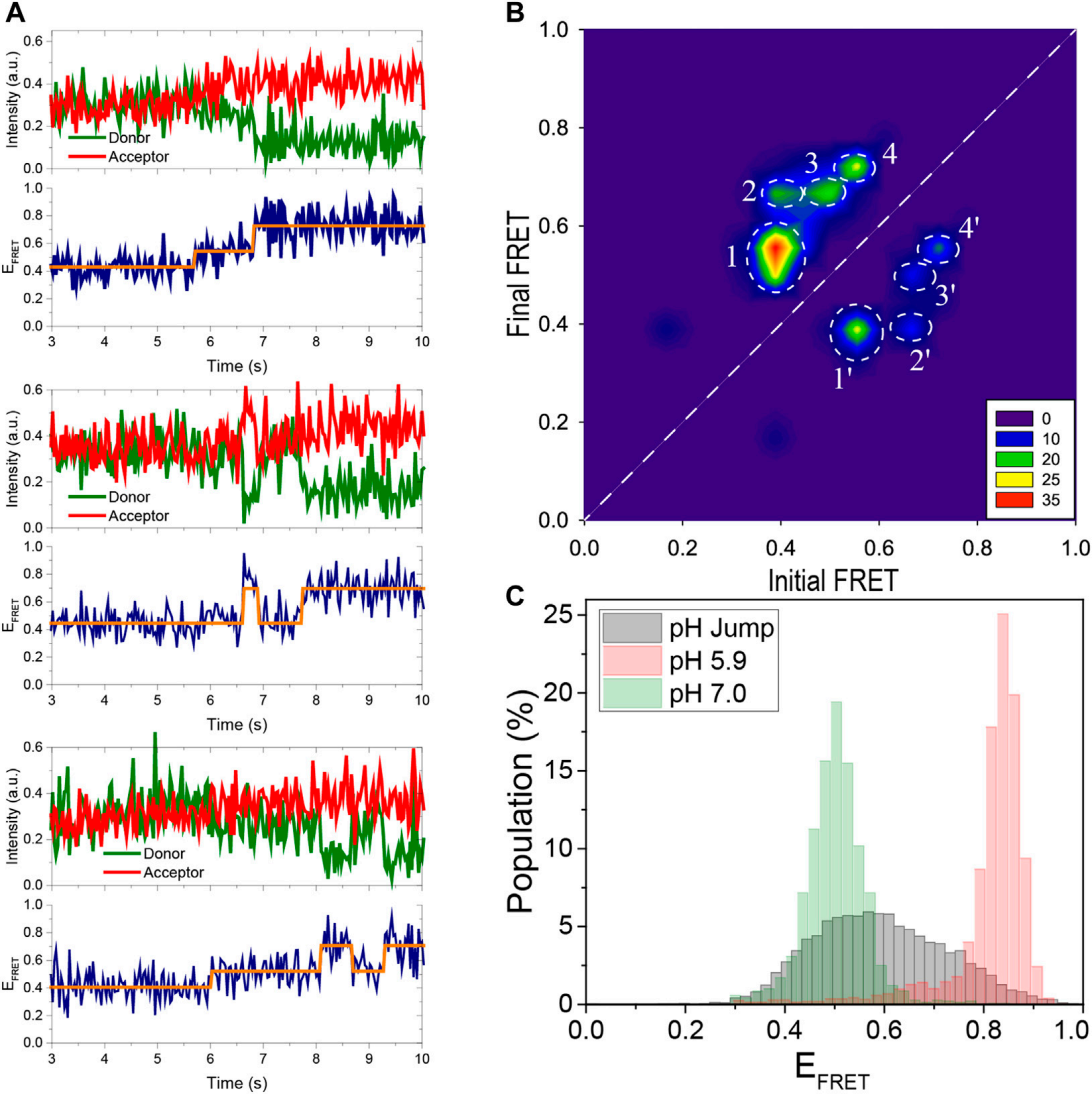
Using pH jump measurements, the folding process is monitored in real time. **(A)** Three representative FRET time traces are shown. As the pH is decreased from 7.0 to 5.3, the DNA molecules convert from an unfolded to a folded state. The traces show distinct intermediate states that were detected using HMM analysis (the orange lines overlaid on the data). As shown in the middle and bottom traces, the i-motif occasionally transitions from a folded state to the unfolded state (middle trace) or to an intermediate state (bottom trace) before refolding. **(B)** A contour plot of the transitions from 105 molecules shown in the TDP. The notable transitions are denoted by white circles and are numbered 1, 2, 3, and 4 in the folding direction. The reverse of these transitions in the unfolding direction are numbered 1′, 2′, 3′, and 4′. All folding and unfolding processes involve intermediate states between the completely unfolded E_FRET_ ≈ 0.40 and completely folded E_FRET_ ≈ 0.75 states. **(C)** The gray histogram is constructed from the FRET levels spanning the interval 2 s before and 2 s after the transition. This representation emphasizes the contribution of intermediate states that are absent in steady-state histograms obtained 30 min after the buffer exchange. For comparison, the steady state distributions at pH 7.0 (completely unfolded) and pH 5.9 (completely folded) are depicted with green and red histograms, respectively, as a reference.

**FIGURE 5:**
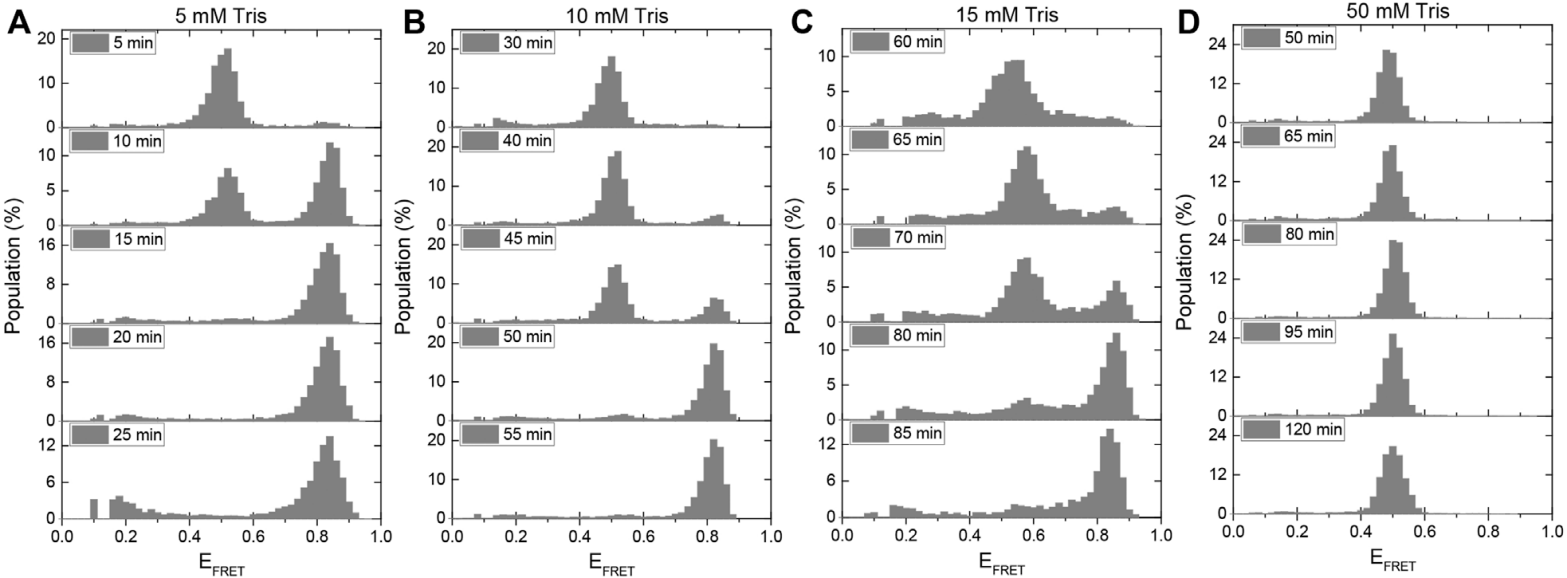
A method for using the i-motif as an *in-situ* pH sensor in smFRET experiments has been developed. When gloxy is used as the oxygen scavenging system, the gradual acidification of the environment is quantified for **(A)** 5 mM Tris, **(B)** 10 mM Tris, **(C)** 15 mM Tris, and **(D)** 50 mM Tris. The amount of time required for the pH to drop from 7.5 to 6.5, at which point i-motif folding begins, increases as the buffer strength is increased. In the presence of 50 mM Tris, all of the DNA molecules remain unfolded for the entire 120-min imaging period.

Bulk biophysical methods were extensively used to investigate the folding and stability of the i-motif as a function of pH and ionic conditions. (Liedl and Simmel, 2005; Nguyen et al., 2017; Assi et al., 2018; Brown and Kendrick, 2021). However, limited work has been done using single molecule methods (Choi et al., 2011; Kim et al., 2012; Selvam et al., 2017; Jonchhe et al., 2018; Megalathan et al., 2019). In this study, we investigate the stability, intermediate folding states, and evolution of i-motif formation at different pH and ionic conditions using single molecule Förster resonance energy transfer (smFRET) microscopy. After introducing the construct, method, and proof-of-principle measurements that demonstrate the stability of pH in the protocatechuic acid (PCA) and protocatechuate-3,4-dioxygenase (PCD) oxygen scavenging system (PCA/PCD) in Figure 1, we show the capabilities of our approach in Figures 2, 3, clearly identifying the i-motif structure and evolution of its folding over a broad pH and salt concentration range. Using this approach, we monitor the folding of i-motif in real time in Figure 4. These results showed at least one prominent intermediate folding state is present during the most of folding events. Figure 5 shows proof-of-principle measurements demonstrating the capability of i-motif structure as an *in-situ* pH sensor for smFRET studies. In this measurement, we show that when “gloxy” (glucose oxidase plus catalase), the most commonly used oxygen scavenging system for single molecule fluorescence studies, is used to improve dye photostability, the i-motif structure can detect the time-dependent drop in pH of the environment.

## Materials and methods

### DNA constructs

The following HPLC purified, biotin conjugated, and fluorescently labeled DNA oligonucleotides were purchased from Integrated DNA Technologies (Coralville, IA, United States):

Stem Strand:/Biotin/GCCTCGCTGCCGTCGCCA-Cy5

i-motif Strand: Cy3-TTCCCTAACCCTAACCCTAACCCTTTGGCGACGGCAGCGAGGC

The nucleotides underlined form the i-motif structure. A partial duplex DNA (pdDNA) construct was prepared by annealing a short stem strand, with biotin at 5′-end and Cy5 at 3′-end, and a long strand that contains a complementary sequence to the stem and the i-motif forming C-rich sequence. The two strands were heated at 90°C for 3 min followed by cooling to room temperature over 3 h. The annealing reaction was performed in 150 mM KCl and 10 mM 2-ethanesulfonic acid (MES, at pH 7.0).

### Sample preparation and smFRET assay

Laser-drilled quartz slides and glass coverslips were thoroughly cleaned with potassium hydroxide (KOH) and acetone before piranha etching, and NHS-ester polyethylene-glycol (PEG) passivation. Surface passivation was accomplished using a PEG mixture with a 100:2 ratio of m-PEG-5000: biotin-PEG-5000 (Laysan Bio Inc.). The PEG prevents non-specific binding of DNA molecules to the surface, whereas biotin-PEG-5000 provides an attachment point for biotinylated DNA molecules that connect to the biotin-PEG *via* neutravidin. The sample chambers were then prepared by sandwiching double-sided tape between these PEGylated slides and coverslips. After extensive washing with water, 0.01 mg/ml neutravidin was introduced into the chamber and incubated for 15 min. Biotin-tagged pdDNA constructs were then introduced to chamber and immobilized on PEG surface *via* biotin-neutravidin attachment.

The pdDNA samples (annealing at 1 µM concentration) were diluted to 50 pM in multiple steps and incubated in the sample chamber for 3–5 min at 150 mM KCl and 10 mM MES (pH 7.0). To remove excess pdDNA molecules that did not bind to the surface, the chamber was washed with 10 mM MES (pH 7.0). This procedure yielded approximately 250–280 molecules per imaging area of 4 μm^2^× 10^3^ μm^2^. Except for the pH jump experiments shown in Figure 4, 20 short movies of 15 frames/movie were recorded at a frame acquisition rate of 100 ms/frame for each assay condition. For pH jump experiments where folding intermediates were studied, longer movies of 2000–4,000 frames/movie at 31 ms/frame were recorded.

For our measurements, two distinct imaging buffers were used. Except for the data shown in Figure 4, the imaging solution contained 50 mM MES of indicated pH, 2 mM Trolox, 25 mM PCA, 0.35 mg/ml PCD, 0.1 mg/ml bovine serum albumin (BSA), 2 mM MgCl_2_, and indicated concentrations of KCl. This solution will be referred to as PCA/PCD imaging buffer. Trolox increases brightness of the fluorophores by quenching their dark triplet state. PCA and PCD form an oxygen scavenging system that delays photobleaching of fluorophores. BSA patches the surface areas that might have imperfect PEGylation. The imaging solution was incubated with the DNA molecules in the channel for 30 min before measurements in order to allow the i-motif structures to reach steady-state and PCA/PCD to reduce the oxygen concentration to adequate levels. We made sure the pH was stable to three decimal points when preparing the buffers, but we rounded them to the nearest one decimal point for reporting clarity, so pH 6.324 was reported as 6.3. However, to demonstrate the variation between different conditions within the uncertainties of the measurement (±0.05 or less in pH), we reported the equilibrium constants to two decimal points, e.g., 6.32.

Instead of the PCA/PCD system, glucose oxidase and catalase (also known as gloxy) were used as the oxygen scavenging system for the pH jump measurements shown in Figure 4. All other contents of the imaging buffer were otherwise same as listed above. This solution will be referred to as gloxy imaging buffer. When compared to PCA/PCD, gloxy has a significant advantage in that long incubation times are not required to remove oxygen, and data acquisition can start as soon as the imaging buffer is introduced into the chamber. This allows for real-time observation of the i-motif folding because movies could be recorded while the pH of the environment was reduced from 7.0 to 5.3 *via* a buffer exchange [referred to as a pH jump in this study and “flow measurement” in others where structural changes in DNA were recorded in real time (Ray et al., 2013)]. For the pH jump measurements, the DNA molecules were first incubated in a PCA/PCD imaging buffer containing 50 mM MES at pH 7.0 and 150 mM KCl. After 30 min of incubation, a long movie of 2000–4,000 frames was started recording at an acquisition rate of 31 ms/frame. After recording ∼200 frames, a gloxy imaging buffer containing 50 mM MES at pH 5.3 (all other ingredients were kept identical) was flown into the sample chamber while the recording continued.

Despite the fact that gloxy facilitated the pH jump experiments, it is known to cause a gradual pH drop in the chamber due to the generation of gluconic acid as a by-product of reducing the free oxygen concentration as per the following reaction:

$$\beta -D-glucose+O_{2}+H_{2}O\overset{glucose\;oxidase}{\to }gluconic\;acid+H_{2}O_{2}$$

$$H_{2}O_{2}\overset{catalase}{\to }H_{2}O+\frac{1}{2}O_{2}$$

The rate of pH drop is determined by buffer strength, which we measured in Figure 5 using the i-motif as an *in-situ* pH sensor. These measurements began at physiological pH (7.5) which motivated the use of Tris-HCl as the buffer rather than MES due to its higher pKa (Good et al., 1966). For these measurements, the imaging solution contained the following: Indicated concentration of Tris-HCl (initial pH 7.5), 0.1 mg/ml glucose oxidase, 0.02 mg/ml catalase, 2 mM Trolox, 0.1 mg/ml BSA, 2 mM MgCl_2_, and 150 mM KCl.

### Imaging setup

The smFRET measurements were performed using a prism-type total internal reflection fluorescence microscope equipped with an Olympus IX-71 microscope and an Andor IXON EMCCD camera (IXON DV-887 EMCCD, Andor Technology, CT, USA-now part of Oxford Instruments). A green laser beam with a wavelength of 532 nm (Spectra Physics Excelsior) was used to excite the donor fluorophore. The fluorescence signal was collected with an Olympus water objective (60×, 1.20 NA).

### Data analysis

The recorded movies were analyzed and time traces of donor intensity (I_D_) and acceptor intensity (I_A_) for each molecule were generated using a custom software written in C++. The FRET efficiency (E_FRET_) was calculated using $E_{FRET}=I_{A}/\left(I_{A}+I_{D}\right)$ . The FRET efficiency population histograms were constructed from single molecule traces such that each molecule contributed equally, regardless of how long the molecules remained fluorescent (time it took for molecules to photobleach). To correct for the donor signal leakage into the acceptor channel, which results in the donor-only (DO) peak, the leakage was subtracted from the histogram and the DO EFRET level was shifted to zero.

We used a previously published method to investigate the pH and salt concentration dependence of the i-motif structure (McKim et al., 2016). Accordingly, an equilibrium constant *K* is defined as:

$$K=\frac{folded\;population}{unfolded\;population}=\frac{fraction\;i‐motif}{1-\left(fraction\;i‐motif\right)}$$

The pH or salt concentration where folded and unfolded populations are equal, the equilibrium constant K=1 , will be referred to as $K_{eq}.$ We plot log(K) vs. pH or log(K) vs. log(salt concentration) , and linearly fit the most rapidly varying segments of the curve to determine $K_{eq}$ . When K=1, log(K)=0 , so the pH or salt concentration where the line crosses 0 corresponds to corresponding ${\text{K}}_{\text{eq}}$ . The slope of log(K) vs. pH has been interpreted as the number of protons gained or lost during the folding or unfolding transitions, respectively (McKim et al., 2016). These analyses were carried out using the Origin software program. The errors in the cited $K_{eq}$ values are the standard deviations obtained from the fitting analysis.

## Results and discussion

### Stability of pH in PCA/PCD oxygen scavenging systems

In this study, we used two different oxygen scavenging systems in the imaging buffer: PCA/PCD and gloxy (glucose oxidase + catalase). Both have unique characteristics that make them better choices for specific applications. The PCA/PCD provides a constant pH over multiple hours of imaging; however, it is typically necessary to incubate PCA/PCD in the sample chamber for 10–30 min before it becomes effective. On the other hand, the gloxy system accumulates gluconic acid in the imaging chamber over time, gradually lowering the pH. Because of these characteristics, the majority of measurements in this study were performed with PCA/PCD since maintaining a stable pH was critical to avoiding a compounding factor when the salt or pH of the environment was changed. Since we were primarily interested in the steady state distributions, we were able to incubate PCA/PCD in the chamber for 30 min before each measurement. However, gloxy was preferred as the oxygen scavenging system in pH jump measurements, where the folding process was much faster and we were interested in the folding intermediates during this fast process.

A schematic of the DNA construct with the donor-acceptor dye positions is shown in Figure 1A. Figure 1B shows an example smFRET time trace demonstrating a transition from the unfolded to the folded state where the FRET efficiency rises from E_FRET_ ≈ 0.5 to E_FRET_ ≈ 0.9. As a proof of principle measurement, we measured the stability of pH in PCA/PCD oxygen scavenging system over a 120-min imaging period in 150 mM KCl. We chose a pH value where even minor changes in pH would result in detectable changes in the fraction of the folded i-motif population as we expect pH to be relatively stable in PCA/PCD system. As per data in Figures 2C,D, pH 6.3 would be the best choice for this purpose since approximately 50% of the molecules are folded and small changes in pH would result in significant changes in the folded fraction. For these studies, we also used MES buffer as it is a better buffer that pH range. Figure 1C shows the overlaid FRET histograms at initial pH of 6.3 after 30 and 120 min incubation in imaging buffer containing PCA/PCD. The histograms are essentially identical, indicating that the pH (6.29 ± 0.05) in PCA-PCD system remains remarkably stable over 120 min imaging period.

### Stability of i-motif as a function of pH

Figure 2 shows smFRET population histograms obtained from pH titration (pH 5.9–7.0) at 20 mM (Figures 2A,B), 150 mM (Figures 2C,D), and 300 mM KCl (Figures 2E,F). The high FRET states (E_FRET_>0.70) in these histograms represent the folded i-motif, while the low FRET states (E_FRET_<0.60) represent the unfolded state. As pH decreases, the population of the folded state increases, while the population of the unfolded state decreases, as expected. All molecules were folded at pH 5.9 (a single peak at E_FRET_ ≈ 0.80) and unfolded at pH 7.0 (a single peak at E_FRET_ ≈ 0.50) for all three KCl concentrations. The entire population switches from i-motif to random coil between pH 6.0 to 6.6 in agreement with a previous report (Liedl and Simmel, 2005).

Figures 2B,D,F show the steady state variation of folded state as a function of pH for 20 mM KCl, 150 mM KCl, and 300 mM KCl, respectively. The linear fits (green lines in the insets of the cited figures) to log(K) vs. pH data were used to calculate the pH equilibrium constants (K_eq_) where 50% of the molecules are folded. These fits yielded K_eq_ = 6.38 ± 0.05 at 20 mM, K_eq_ = 6.23 ± 0.05 at 150 mM, and K_eq_ = 6.17 ± 0.05 at 300 mM KCl. The uncertainties were based on the pH range which results in a 5% variation in the fraction of the i-motif population. These results are consistent with earlier reports that showed a lower pKa for cytosine at higher NaCl or KCl concentrations (Mergny et al., 1995; Nguyen et al., 2017). Therefore, at a given pH value, cytosine is less likely to be protonated at a higher KCl concentration, which results in a lower stability i-motif.

Our observed equilibrium constants (for example K_eq_ = 6.23 ± 0.05 at 150 mM KCl) are higher than those observed in some bulk measurements while they are consistent with others. Using circular dichroism, Nguyen et al. observed that K_eq_ ≈ 5.75 at 165 mM and K_eq_ ≈ 5.85 at 115 mM KCl, which can be interpolated to K_eq_ ≈5.8 at 150 mM KCl (Nguyen et al., 2017). On the other hand, Lannes et al. reported pH = 6.26 as the point where 50% of telomeric i-motif molecules are folded at 50 mM KCl (Lannes et al., 2015), while Ding et al. reported pH = 6.1 for the corresponding point at 1 M KCl (Ding et al., 2015), and Wright et al. reported pH = 6.31 for 100 mM NaCl (Wright et al., 2020). These values are overall consistent with the values we report and demonstrate the range of values observed in different assays, probably due to variations in the specifics of the assay conditions and the way the samples are prepared. On a related note, a surface-induced stabilization of the i-motif was reported (Adam et al., 2018), which might also affect the results of measurements that localize the i-motif on the surface compared to bulk measurements.

### Stability of i-motif as a function of ion concentration

We also investigated the formation and stability of i-motif structures as a function of (KCl) and (NaCl) at a fixed pH. Figures 3A,B show the titration of (KCl) from 0–1,000 mM while pH was fixed at 6.3. As (KCl) increases, the unfolded state population increases, as expected. In agreement with previous studies, the system could be driven from nearly completely folded to nearly completely unfolded between 80 and 400 mM KCl (Liedl and Simmel, 2005). As shown in Figure 3A, as (KCl) increases, unfolded state peak gradually shifts to higher FRET values. This shift in the FRET level of the unfolded state is due to better shielding of the negatively charged ssDNA backbone at higher salt concentrations. As a result, the folded and unfolded peaks approach each other since the shift in the folded state is significantly less prominent, which is expected given that unstructured ssDNA forms only a small fraction of the overall construct (two spacer nucleotides on either side of the i-motif). This could make smFRET less sensitive to i-motif formation at very high salt concentrations (several molar), but this is not the case until 1 M KCl. The formation of i-motif requires very low pH at higher (several molar) salt concentrations where, in our experience, the commonly used photostability of fluorophores in smFRET measurements is typically poor. As a result, studying the structure and kinetics of i-motif using smFRET at such high salt concentrations would be challenging. The evolution of the fraction of i-motif population as function of (KCl) is shown in Figure 3B. The linear fit to the most rapidly varying segments of the curve of log(K) vs. log([KCl]) is shown in the inset of Figure 3B as a green line, which yielded an equilibrium constant K_eq_ = 215 ± 3 mM KCl at pH 6.3. We performed similar salt titration measurements using NaCl (Figures 3C,D) because the i-motif is closely related to GQ, and the GQ stability is strongly dependent on the ion type (Tran et al., 2011). Unlike the GQ, we found that i-motif stabilities is very similar in K^+^ and Na^+^, with K_eq_ = 198 ± 3 for NaCl.

### Real time monitoring of i-motif folding

We performed buffer exchange measurements in order to observe folding of the i-motif in real time. In these measurements, a buffer at a lower pH (pH 5.3) was injected into the sample chamber containing unfolded molecules at a higher pH (pH 7.0). Gloxy was used as the oxygen scavenging system in these measurements rather than PCA/PCD, which was used for the data in Figures 2, 3. This allowed us to image the folding process in real time, which occurs within a few seconds of the buffer exchange. A syringe pump was used to inject the lower pH buffer into the chamber, minimizing system disruption and allowing continuous imaging of the folding process. Three representative smFRET time traces capturing folding process are shown in Figure 4A. We performed a Hidden–Markov modeling-based analysis (vbFRET) (Bronson et al., 2009) on traces that showed transitions to determine the different folding states in a bias-free manner. Examples of this are shown in Figure 4A where orange lines are overlaid on the FRET data. The traces show intermediate folding state in between unfolded and folded state, in addition to occasional unfolding events before the molecule transitions to stable folded state.

The transition density plot (TDP) constructed from 105 such traces is shown in Figure 4B. The *x*-axis in TDP represents the FRET state before the transition, known as initial FRET, and the *y*-axis represents the FRET state after the transition, known as final FRET. The number of transitions is represented by the color in TDP, with red being the most frequently occurring and blue being the least. A folding transition from E_FRET_ ≈ 0.40 to E_FRET_ ≈ 0.70, for example, will contribute as one unit to TDP at point (0.40, 0.70). Similarly, an unfolding transition from E_FRET_ ≈ 0.70 to E_FRET_ ≈ 0.40 will contribute as one unit to TDP at point (0.70, 0.40). The white dashed line indicates the 45°. Transitions above this line are due to folding while those below it are due to unfolding. Since a higher pH buffer was swapped for a lower pH buffer and the transitions were biased in the folding direction, the levels above the 45°-line are more populated. The states below the 45°-line, however, are clearly detectable, indicating that the folding process is not irreversible and that some molecules occasionally unfold during this transition period.

In TDP, we observed a prominent intermediate state, which we were unable to detect in the previous steady state measurements. In the folding direction, the distinct transitions are marked by white dashed circles numbered 1, 2, 3, and 4. These transitions in the unfolding direction are marked by 1′, 2′, 3′, and 4′, respectively. As shown in the TDP, the most populated transitions are from E_FRET_ ≈ 0.40 to E_FRET_ ≈ 0.55 (circle 1). The molecules that reach this intermediate state at E_FRET_ ≈ 0.55 continue to fold and reach either E_FRET_ ≈ 0.70 (Circle 3) or E_FRET_ ≈ 0.75 (Circle 4) state. There are also molecules that made a direct transition from the unfolded state at E_FRET_ ≈ 0.40 to the folded state at E_FRET_ ≈ 0.70 (Circle 2). All of these transitions are reversible, as indicated by the circles 1′, 2′, 3′, and 4′. Assuming that the E_FRET_ ≈ 0.75 state is completely folded and the E_FRET_ ≈ 0.40 state is completely unfolded, no direct folding or unfolding events are observed, i.e. there are no transitions around (0.40, 0.75) or (0.75, 0.40). Instead, during both folding and unfolding process, the molecules first transition to the intermediate state before unfolding or folding completely. Since the intermediate state is relatively short-lived and only observed during transitions, it is not surprising that it was not detected in steady state histogram shown in Figures 2, 3, which survey the molecules after they have been incubated in respective buffers for about 30 min. To amplify the contribution of the intermediate populations in the FRET histograms, we constructed a new histogram from the time traces of these pH jump measurements, including only the segments spanning 2 s before and 2 s after the transition. Figure 4C shows the new histogram, which shows that the states between the unfolded (E_FRET_ ≈ 0.40) and folded (E_FRET_ ≈ 0.75) are significantly populated (gray histogram). We overlaid the corresponding steady state histograms at pH 5.9 (red histogram) and at pH 7.0 (green histogram) for reference.

### I-motif as an *in-situ* pH sensor

Small organic fluorophores are used in single molecule fluorescence studies, which photobleach (a chemical transition of the fluorophores to a non-fluorescent state) primarily due to free oxygen radicals. Hence, the presence of the oxygen scavenging system in the imaging buffer is critical for single molecule fluorescence studies. The two most commonly used oxygen scavenging systems are gloxy and PCA/PCD. Even though fluorophore brightness and lifetime have been shown to be superior in PCA/PCD compared to gloxy for some commonly used fluorophores (Lee et al., 2010), gloxy is the most widely used oxygen scavenging system in single molecule fluorescence measurements due to its fast action, with a downside of progressively decreasing pH in the sample chamber. As a result, understanding the extent of the pH drop in the presence of gloxy is critical for establishing the time limits for a relatively constant pH in the environment (more accurately time limit before pH drops below an acceptable threshold value).

Previously, a ratiometric dual emission dye SNARF-1 was used to investigate this important question (Shi et al., 2010). The pH variation within the sample chamber was determined by characterizing the relative populations of the two peaks at different pH values. The sample chamber was placed in the path of the light beam within the spectrofluorometer and the pH was determined using bulk FRET signal. In our study, the pH variation was monitored based on folding of the i-motif. Because the pH drop depends on the strength of the buffer, we repeated the measurement in 5 mM, 10 mM, 15 mM, and 50 mM Tris with an initial pH of 7.5. We expect the pH drop in weaker buffers to be faster, so the initially unfolded DNA strands should fold into the i-motif after a shorter incubation period than in stronger buffers. We conclude that the pH of the environment should be 5.9 for all DNA molecules to fold into the i-motif based on the ionic conditions of this assay (150 mM KCl) and the calibration data presented in Figures 2C,D.

Figures 5A–D shows the change in the population of unfolded molecules over the time after adding gloxy to the chamber at different Tris concentrations. All the DNA molecules fold into i-motif after incubation of the molecules in 5 mM, 10 mM, and 15 mM Tris for approximately 15, 55, and 85 min, respectively. Under these buffer conditions, these observations establish a time scale for pH to drop from 7.5 to around 6.5, where i-motif population becomes detectable. In 50 mM Tris, the DNA molecules remain completely unfolded after 120 min of imaging. This implies that the pH in 50 mM Tris remains above 6.5 (as per Figures 2C,D) even after 120 min of incubating the gloxy in the chamber. These findings are quantitatively consistent with the pH drop observed in ratiometric bulk FRET measurements on SNARF-1 (Shi et al., 2010). It should be noted that using lower concentrations of glucose oxidase in the imaging buffer can slow down the pH drop; however, this is often accompanied by lower photostability. In our observation, reducing the concentration of glucose oxidase by a factor of ten results in a two-fold reduction in Cy3/Cy5 photostability while maintaining fluorophore brightness.

Even though the proposed approach does not report the variation of the pH from 7.5 to 6.5, it does provide the time frame over which the pH remains approximately within the physiological range. For example, in 10 mM Tris, the pH drops from 7.5 to 6.5 in about 35 min (approximate time when folded population first appears) and to 5.9 in about 55 min (when all molecules transition to folded state). Therefore, if the acceptable pH range for a given measurement is 7.5–6.5, the experimenter should refresh the imaging buffer within 30–40 min (Figure 5B). It might be possible to establish better guidelines by testing different models for the rate of pH drop over time. The simplest of these models is to assume the pH drops at a constant rate (linearly) within the range of interest (approximately 7.5–6.0). Using the time points for pH 7.5 and 6.5 and making a linear approximation, we would estimate the pH drops at a rate of (7.5–6.5)/(35 min) = 0.029 min^−1^, where 35 min is taken as the mid-point between 30 (where we do not observe any folded population in Figure 5B) and 40 min (where a significant folded population is observed). Similarly, if we use the time points for pH 7.5 and 5.9 as reference points, we would obtain a pH drop rate of (7.5–5.9)/(55 min) = 0.029 min^−1^. The consistency of the two numbers suggests the simple linear approximation of pH drop over time might be acceptable over this pH range (6.0–7.5) and buffer strength, which are commonly used in many single molecule fluorescence measurements. Assuming these rates, we would estimate the pH drops from 7.5 to 7.0 in about 17 min in 10 mM Tris. An important aspect of this method is that it reports the pH as experienced with surface immobilized molecules, which is how most experiments that utilize total internal reflection fluorescence (TIRF) microscopy.

## Conclusion

The findings of this study demonstrate that smFRET can be used to study i-motif systems under a wide range of ionic and pH conditions with proper DNA construct design and fluorophores placement. Our different image acquisition approaches clearly identify steady state distributions, dynamic transitions, and intermediate states during the folding process. This versatile smFRET method could also be used to investigate the interactions of i-motif structures with proteins, small molecules, or competing nucleic acid sequences. The i-motif sequence we investigated was highly sensitive to pH variations in 6.3 ± 0.3 range. Based on this observation, we demonstrated the feasibility of using the i-motif as an *in-situ* pH sensor in smFRET experiments to determine the time limits at which the pH of the system falls below the physiologically relevant range.

## Data Availability

The original contributions presented in the study are included in the article, further inquiries can be directed to the corresponding author.
